# “Sunlight-driven catalytic degradation of MB dye and multi-cycle Re-usability analysis of Cu_2-x_Se nanoparticles”

**DOI:** 10.1016/j.heliyon.2024.e39669

**Published:** 2024-10-22

**Authors:** Pushpanjali Patel, Rekha Garg Solanki, Prerna Gupta, KM Sujata

**Affiliations:** Department of Physics, Dr. Hari Singh Gour University Sagar, M.P, 470003, India

**Keywords:** Cu_2-x_Se NPs, Photo-catalysis, First and second order kinetics, Re-usability etc

## Abstract

One of the best ways to remove organic dyes based contaminant from water resources and industrial waste water is the sunlight driven photo-catalytic degradation method. The theme of the present investigation is the photocatalytic degradation of methylene blue (MB) dye using economically produced Cu_2-X_Se nanoparticles (NPs) catalyst under solar radiations. The Cu_2-X_Se NPs crystallized in the cubic structural phase with an average crystallite size around 19 nm. The direct band gap was found to be 2.1 eV. The PL spectra and corresponding CIE diagram show the Cu_2-x_Se NPs emitted yellow color. The SEM micrographs show that the small grains staked over others to form large grains or patches. The FTIR and EDX spectra confirmed the formation of Cu_2-X_Se NPs. The obtained optimum photocatalytic degradation efficiency for Cu_2-X_Se NPs is 90.3 %. For first cycle analysis. The pseudo-first order and second order models were used to analyze the kinetic data of Cu_2-X_Se NPs for varying concentrations. The multiple-cycle degradation analysis by catalyst of MB dye in one day spam for continuous four cycles were also analysed and discussed. The sun light driven multi-cycle catalytic degradation confirms the re-usability of Cu_2-x_Se NPs for the treatment of industrial waste water and other contaminated water bodies for the survival of aquatic life and for saving environment.

## 1 Introduction

Organic pollutants derived from industrial waste are causing one of the world's major environmental disasters and crises, with a high annual cost for removing these pollutants [[1], [2], [3]]. One of the best and the most cost-effective methods to remove these pollutants is photocatalytic activity. A good photocatalytic material should be non-hazardous, cheap, and highly effective in utilizing solar radiations [4]. During the last two decades, semiconductor materials have been extensively used in photo catalysis of organic dyes. So far, different nano-structured materials like TiO_2_ (3.2 eV), ZnO (3.63 eV), and SnO_2_ (1.7–3.1 eV) nanoparticles have received considerable attention due to their higher surface areas than bulk materials and have been significantly investigated for both energy and environmental applications. However, widely used TiO_2_ and SnO_2_ are uneconomical for large-scale production for water treatment. Cu_2-X_Se NPs has been found to be an effective alternative in comparison to TiO_2_ and SnO_2_ nanomaterials due to its high degradation efficiency [[5], [6], [7], [8], [9], [10], [11], [12], [13], [14], [15]]. Cu_2-X_Se NPs is one of the inorganic materials in the family of I–VI semiconductors and shows both direct (2.1–2.27 eV) and indirect (1.1–1.7 eV) band gaps and utilized UV and Visible spectrum of solar radiations for dye degradation even without the use of reducing agents such as NaOH and H_2_O_2_ during catalysis process [[16], [17], [18], [19]]. Copper selenide is a unique metal chalcogenide that exists in many structural forms with different stoichiometries such as Cu_2_Se, α-Cu_2_Se, Cu_3_Se_2_, Cu_2_Sex, CuSe_2_, CuSe, and Cu_7_Se_4_ as well as non-stoichiometric forms such as Cu_2-X_Se and can be constructed into several crystallographic forms (monoclinic, hexagonal, tetragonal, and cubic) [[20], [21], [22], [23], [24], [25]]. In addition to this, copper selenide shows various applications in the fields of catalysts, solar cells, sensors, electronic devices, and anti-bacterial activity. It has been synthesized in well-defined nano-structures with various morphologies, such as nano-dendrites [26], nano-crystals [27], nano-structures [28], nano-plates [28], [29], nano-spheres [30], nanowires [31], and nano-rods [32]. Various synthesis method under different preparation conditions were employed for synthesis of copper selenide NPs as hydrothermal [33], solvothermal [34], chemical bath deposition method [35], sonochemical [36], and co-precipitation method [37].

Yanjie et al. synthesized hexagonal CuSe nano-flakes by hydrothermal method. They predicted degradation of MB dye in the presence of sunlight [19]. Wenwan et al. synthesized Cu_3_Se_2_, Cu_2_Se & Cu_2_O hollow microspheres by sacrificial template method. They reported the photo-catalytic activity by Cu_3_Se_2_, Cu_2_Se & Cu_2_O on methyl blue solution (50 mg/L) in the presence of uv–visible radiation. Cu_3_Se_2_, Cu_2_Se & Cu_2_O exhibit excellent photo-degradation activities up to 90 % [21]. Sonia et al. synthesized CuSe NPs by reflux condensation method and predicted the photocatalytic degradation efficiency of CuSe NPs against, Methylene blue (MB) and Rhodamine-B (RhB) to be 76 % and 87 %, respectively [38]. Wang et al. synthesized hexagonal CuSe nano plates and Cu_2_Se NPs by one-pot solution method. They reported the photocatalytic degradation of MB dye under UV–Visible light [39]. K. Kaviyarasu et al. synthesized CuSe NPs by two step hydrothermal method. They reported that the photodegradation of MB Dye was 74 % in aqueous solution under visible light [40]. Boding et al. synthesized hexagonal Cu_2_Se nano-sheets by hydrothermal method. They reported the degradation efficiency of Cu_2_Se nanosheets and photo-catalytic activity against MB dye under visible light irradiation in the presence of H_2_O_2_ [41]. The comparison of the above reports in terms of synthesis method, used chemicals, synthesized copper selenide NPs and reducing agents during catalysis of copper selenide were shown in Table 1.

**Table 1:** The synthesis methods, **Precursors** and corresponding compositions/structures of Cu_2-x_Se NPs.

S. No.	Synthesis Method	Precursor and Additional Used	Composition	Reference
1	Co-Precipitation Method	Se, Cu, Hydrazine Hydrate	Cu_2-X_Se	This work
2	Hydrothermal method	CuCl, NaOH, PVP, Se, H_2_O_2_	CuSe Hexagonal	[19]
3	Facial Sacrificial Template Method	NaOH, CuCl_2_, ascorbic acid, Na_2_So_3_, NaOH, Se, H_2_O_2_	Cu_3_Se_2_/Cu_2_Se/Cu_2_O hollow microsphere	[21]
4	Reflux Condensation Method	CuCl_2_.H_2_O, Ethylene glycol, Se, Hydrazine Hydrate	CuSe	[38]
5	One-Pot approach	CuCl, SeO_2_, Hydrazine Hydrate, ethylene glycol, PVP, cetyltrimethyl ammonium bromide, Triton X-100, sodium dodecyl sulfate, H_2_O_2_	Cu_2_Se	[39]
6	Two step Hydrothermal Synthesis	CuCl_2_, Ethylene Glycol, Se, Hydrazine Hydrate	CuSe	[40]
7	Hydrothermal Method	Se, Cu(CH_3_COO)_2_, NaOH, Beta cyclodextrin, ascorbic acid, H_2_O_2_	Cu_2_Se	[41]

The above reports used various complexing and reducing agent as ascorbic acid, polyvinyl pyrrolidone, triton X-100, cetyltrimethyl ammonium bromide, and sodium dodecyl sulfate, etc. These chemicals are chain based and of high cost and adopted method need specific setups for synthesis; hence the produced CuSe NPs are costly. Among all methods, the co-precipitation process produced highly crystalline materials with high purity, small size distribution, and high surface area to volume ratio in an economic way. The above mention properties help to improve photo-catalytic activity of synthesized materials. We used modified co-precipitation method which is free from chain-based costly chemicals and complex instrumentation.

In the present study, Cu_2-X_Se NPs were synthesized via modified co-precipitation method with the least number of chemicals, at comparative low temperature (i.e., 80^o^C) and the reaction took place in an aqueous medium. Further, the prepared Cu_2-X_Se NPs were used as a catalyst to degrade MB dye with the variation in catalyst concentrations and also with the continuous four cycles in one day time spam for re-usability analysis for 0.05g catalyst (Cu_2-X_Se) concentration. The photocatalytic activities of Cu_2-X_Se NPs were tested in an aqueous medium against MB dye. The photo-degradation process, first and second order reaction kinetics and obtained dye degradation efficiency results were reported and discussed.

## 2 Experimental details

### 2.1 Materials

All the chemical was AR grade and used as-purchased without further purification. Se (Elemental selenium powder, 99.5 %, RANKEM). Cu (Copper metal powder, 99.7 %, HIMEDIA). N_2_H_4_·H_2_O (Hydrazine Hydrate, 99.8 %), Ethanol, 99.9 % and D.I. (De-ionized) water Prepared in laboratory.

### 2.2 Synthesis method

**Fig. 1:**
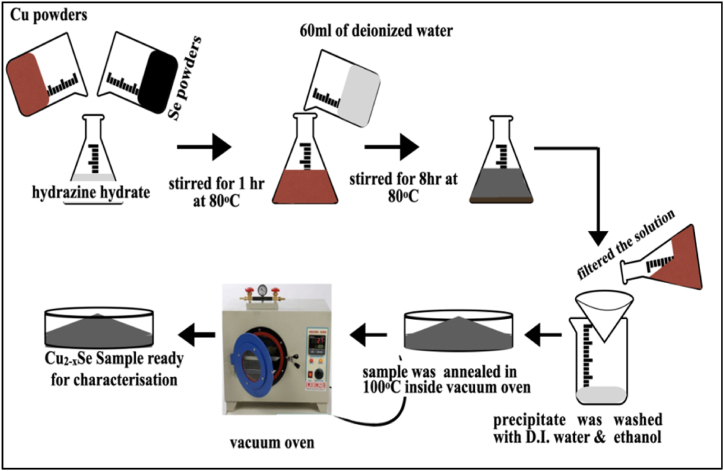
Schematic representation of the synthesis process of Cu_2-X_Se NPs.

Copper selenide NPs were synthesized with a co-precipitation method. Copper and elemental selenium metal powders were used as initial reactants and in equal proportions. Copper and elemental selenium metal powders were added together in 10 ml of hydrazine hydrate and mixed on a magnetic stirrer for 1 h at 80 °C. After that, 60 mL of D.I. water was added. The entire solution was now stirred for 8 h at the same temperature on a magnetic stirrer, maintaining pH at 9. Then the solution was filtered, obtained precipitate was washed with D.I. water many times and finally with ethanol. The obtained precipitate was dried and then annealed at 100 °C in selenium environment for 5 h. The dried sample was gently crumbled and stored for further analysis. The schematic synthesis process of Cu_2-X_Se NPs is shown in Fig. 1.

The Hydrazine hydrate dissociated into hydrazine cation and hydroxiyl anion, and then hydrazine cations react with the copper atoms, oxidized then into Cu^+^ ions and form hydrazine complex. The Cu^+^ ions then react with hydrazine to form a complex in the solution which may be a meta stable complex compound. On the other hand, selenium reacts with hydrazine and form hydrogen selenide. The metastable complex copper compound reacts with hydrogen selenide in the solution for producing copper selenide. The following mechanism for the formation of Cu_2-X_Se NPs have been proposed [[41], [42], [43]]: the possible reactions are shown below as.

**Figure:**
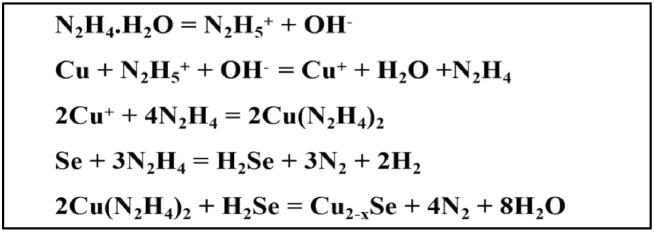

### 2.3 Instruments used for characterization

The crystal structure and phase of Cu_2-X_Se NPs were identified with the help of X-rays powder diffraction instrument (Bruker D-8 Advance machine) of Cu Kα radiation (λ = 0.15418 nm). The range of X-rays spectra was 20° ≤ 2θ ≤ 80° at 30 kV with step size of 0.02°. The Fourier transform infrared spectroscopic instrument (SN 340 Bruker Tensor-37) was used to measure the band vibration over the range from 1200 to 550 cm^−1^ in transmittance mode of operation. Photoluminescence emission spectra (Fluromex Spectrometer from Horiba xenon arc lamp as light source) were measured over wavelength range from 500 nm to 600 nm at an excitation wavelength of 350 nm. The optical absorption spectra, band gap and photocatalytic results were studied at room temperature with a UV–Visible Spectrophotometer (Lab India Analytical UV3092) in the wavelength range 300–800 nm. The morphological features of samples were analysed with a hi-resolution scanning electron microscope (HRSEM) Thermoscientific, EDX attachment with it was used for compositional and elemental mapping results.

## 3 Results and discussion

### 3.1 X-rays diffraction results

**Fig. 2:**
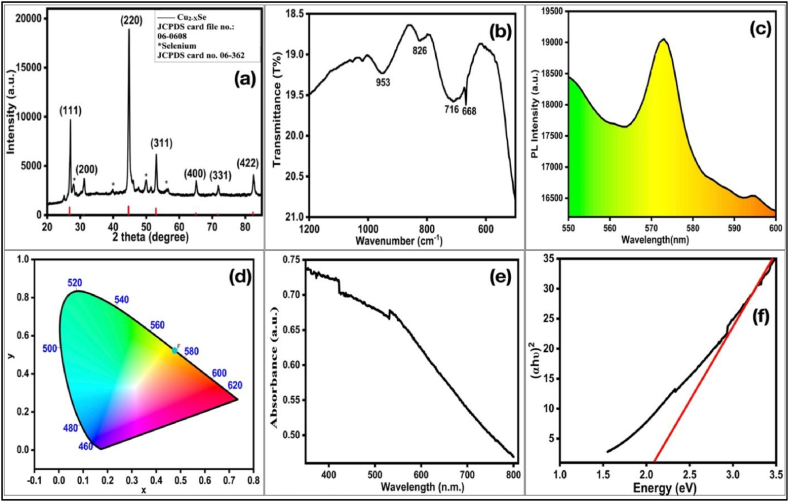
(a) XRD pattern (b) FTIR Spectra (c) The Photo luminescence spectra and corresponding (d) CIE plot (e) UV–vis absorption spectrum & corresponding (f) (αhϑ)^2^ vs (hϑ) plot for Cu_2-x_Se NPs.

Fig. 2(a) illustrates the typical powder X-Rays diffraction (XRD) pattern of Cu_2-X_Se NPs at room temperature for phase identification and crystalline properties. The obtained peaks show compatibility with the diffraction positions of the cubic Cu_2-X_Se phase **where x= 0.15** (mineral Berzelianite phase) according to JCPDS Card No. 06–0608. In the XRD pattern, low-intensity additional peaks of elemental selenium (JCPDS card no. 06–362) were observed. The selenium adsorbed on the surface of sample during annealing in selenium environment which is observed in XRD with low intense peaks. The sharp and narrow peaks with large intensities show that the sample was of good quality. The calculated parameters for all the diffraction peaks were shown in Table 2. The Scherrer’s formula of Eqn. (1) used to calculate the crystallite size [44]. The inter planar distance (d), lattice parameter (a), dislocation density (δ), and micro-strain (*ε*) of Cu_2-X_Se NPs were calculated using Eqn. (2, 3, 4, & 5) respectively [[45], [46]].

Eqn. (1) D=(0.94∗λ)/(β∗cosθ)

Eqn. (2) d=nλ/dsinθ

$$1/d^{2}=\left(h^{2}+k^{2}+l^{2}\right)/a^{2}$$

**Table 2:** Structural Parameters; peak positions(2θ), corresponding Miller planes (hkl), calculated crystallite size, inter planar spacing, micro strain, dislocation density, lattice parameters, unit cell volume, and inter-atomic distance etc.

Miller planes (hkl)	2θ	Crystallite size (nm)	Inter planar spacing d (nm)	Micro strain 10^–4^	dislocation density 10^−3^ (nm^−2^)	Lattice parameter (Å)	Volume of unit cell V(Å^3^)	Inter atomic distances (Å)
111	27.00	21.923	0.329	67.701	2.080	5.712	186.95	Cu-Se = 2.859 Cu-Cu = Se-Se = 4.043 **(Standard:** **Cu-Se = 2.869** **Cu-Cu =** **Se-Se = 4.056)**
200	31.17	15.993	0.286	80.622	3.909	5.730		
220	44.77	17.914	0.202	50.784	3.115	5.710		
311	53.04	17.136	0.172	45.281	3.405	5.718		
400	65.17	18.863	0.142	34.106	2.810	5.718		
331	71.86	21.040	0.131	28.062	2.258	5.719		
422	82.52	17.546	0.116	29.941	3.247	5.719		
**Average**		**18.63**		**48.07**	**2.975**	**5.718**		

Rearrange the above equation.

Eqn. (3) $$a=d\sqrt{h^{2}+k^{2}+l^{2}}$$

Eqn. (4) $$\delta =1/D^{2}$$

Eqn. (5) ε=β/4tanθ

Where λ, θ, β and n are X-ray wavelength (λ = 0.154186 nm) Bragg diffraction angle and line width at half maximum and unity respectively. The volume of the unit cell and inter atomic distances Cu-Se, Cu-Cu & Se-Se for the Cu_2-X_Se NPs were calculated using Eqns. Eqn. (6), Eqn. (7), Eqn. (8).

Eqn. (6) V = a^3^

Eqn. (7) Cu-Se = a/2

Eqn. (8) $$\text{Cu}-\text{Cu}=\text{Se}-\text{Se}=a/\sqrt{2}=0.707a$$

The calculated values from above mentioned parameters and units were given in Table 2. According to Table 2, the obtained average crystallite size was 19 nm. The inter planar spacing were decreases with increase in the peak position. The average lattice parameter was 5.718 Å which nearly matched with the standard value i.e. 5.739 Å. The volume of unit cell was 186.95 Å^3^. The obtained interatomic distances of Cu-Se was 2.859 Å, Cu-Cu & Se-Se were 4.043 Å which was in accordance with the standard data that were shown in Table 2.

### 3.2 Fourier transform infrared spectroscopy results

The Fourier Transform Infrared Spectroscopy (FTIR) spectrum was used to detect the characteristic functional groups associated with the synthesized Cu_2-x_Se NPs as shown in Fig. 2(b). The strong and broad band observed at 953 & 826 cm^−1^, attributed to the stretching vibrations of O–H functional group of water molecules which may be absorbed by the NPs. The absorption peaks at 716 & 668 cm^−1^ were attributed to the bending and stretching vibration of Cu-Se bond, confirmed the formation of Cu_2-x_Se NPs [47].

### 3.3 Photoluminescence results

Photoluminescence (PL) technique is an effective tool to analyze the effect of excited energy on the electronic and optical properties of materials. The PL spectrum of Cu_2-x_Se NPs was recorded at room temperature with an excited wavelength of 350 nm of a Xenon laser source. The obtained PL spectrum in the wavelength range 550–600 nm was shown in Fig. 2(c). It indicated a strong and broad emission peak situated at 573 nm the corresponding band gap value calculated by E = hc/λ was 2.16 eV. The investigation of the emitted color by Cu_2-x_Se NPs, the CIE (International Commission on Illumination)-1931 color chromaticity diagram was plotted and shown in Fig. 2(d). The CIE coordinates located at x = 0.46 and y = 0.52 in the yellow color region; indicated that Cu_2-x_Se NPs are yellow light emitters and found applicable for yellow LEDs and other optoelectronic devices.

### 3.4 UV–visible results

UV–visible spectroscopy is used to identify the optical properties of synthesized Cu_2-X_Se NPs. The absorption spectrum of Cu_2-X_Se NPs was recorded in the wavelength range of 350–800 nm at room temperature and shown in Fig. 2(e). It shows the broad absorption spectra in the scanned wavelength region. The optical band gap was determined by plotting (αhϑ)^2^ vs (hϑ) and shown in Fig. 2(f). The direct optical band gap was obtained from the Tauc’s relation of Eqn. (9) [48].

Eqn. (9) (αhϑ) = A (hϑ - *E*)^n^

Where, A, α, E, and h are a proportionality constant, absorption coefficient, optical band gap energy, and the Planck constant, respectively. In case of Cu_2-X_Se NPs, *n* is taken to be the 1/2 for direct transition. The obtained direct band gap value of Cu_2-X_Se NPs was 2.1 eV, which is in accordance with the band gap obtained from PL peak value analysis. The synthesized Cu_2-X_Se NPs with narrow band suitable for photo catalytic applications [49].

### 3.5 Scanning electron microscope, energy dispersive X-rays & Elemental mapping results

**Fig. 3:**
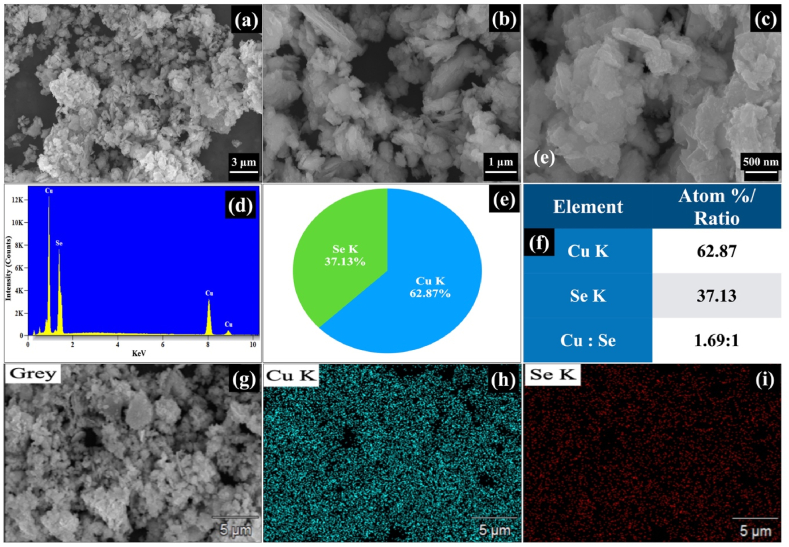
(a–c) SEM micrographs of 3 μm, 1 μm and 500 nm scale (d) EDX spectrum (e) Compositional pie chart (f) elements and their atom ratio (g–i) Elemental mapping images of Cu and Se elements in Cu_2-X_Se NPs.

The SEM micrographs of Cu_2-X_Se NPs of scaling 3 μm, 1 μm and 500 nm were shown in Fig. 3(a–c). It was clearly observed from the micrographs that the irregular-shaped grains were distributed throughout the sample surface. The small grains staked over others to form large grains or patches. The quantitative analysis of constituent elements in the sample was determined using the technique of energy dispersive X-rays analysis (EDX) at room temperature, and the obtained spectrum is shown in Fig. 3 (d). The EDX spectrum confirmed the presence of copper and selenium elements. Fig. 3 (e) shows the compositional pie chart of Cu_2-X_Se NPs. The atomic percentages of constituent Cu and Se elements were 62.87 % and 37.13 %, respectively which was shown in Fig. 3(f). The atomic ratios of copper and selenium (Cu:Se) were 1.69:1, which was confirmed the formation o**f Cu**_**2-x**_**Se (**JCPDS Card No. 06–0608)**, where X = 0.31 instead of X = 0.15.** The selenium element was observed in excess in accordance with the XRD results. The selenium was considered as traces in the sample that significantly affects the photocatalytic process by extending the light absorption range and increase the generation of electron-hole pairs and improving photocatalytic efficiency of prepared samples [40,50,49]. The elemental mapping images were shown in Fig. 3(g–i) and clearly show the uniform spread of constituent elements throughout the sample surface.

### 3.6 Photo-catalytic results with varying Cu_2-X_Se NPs concentrations

**Fig. 4:**
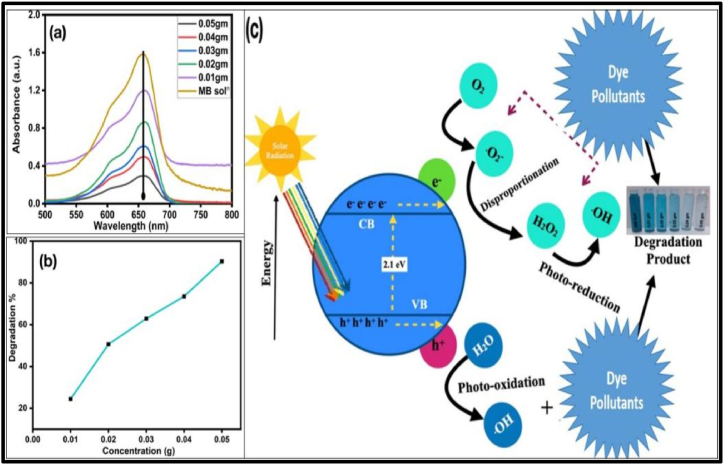
(a) Photo catalytic degradation of MB dye aqueous solutions in exposure of solar radiation (120 min) with varying catalyst concentrations (b) (%) Degradation efficiency with varying Cu_2-X_Se NPs concentration (c) Proposed mechanism of photo catalytic degradation of MB dye via Cu_2-X_Se NPs in presence of solar radiations.

Cu_2-X_Se NPs were appreciated as a catalyst to reduce the contamination of water caused by the colored dyes used in the textile industry. The Cu_2-X_Se NPs have been found to be a powerful catalyst against the majority of organic pollutants. The photocatalytic activity of the prepared Cu_2-X_Se NPs was tested in an aqueous medium with MB dye solution of 1 g/100 ml in the presence of solar radiations at different concentrations of Cu_2-X_Se NPs, such as 0.01, 0.02, 0.03, 0.04, and 0.05 g. The different catalyst concentrations were added separately to 10 ml MB dye solutions filled in test tubes, and then all the test tubes were kept in solar radiation for 120 min. The absorption spectra of MB dye solutions after 120 min' exposure to the sunlight were monitored using a UV–Visible spectrophotometer and the results were shown in Fig. 4(a) and (b). The peak intensities of MB dye solutions decreased as the concentrations of catalyst Cu_2-X_Se NPs increased in the test solutions. According to the Beer-Lambert’s law, decrease in absorbance is directly related to the concentration. Hence, the decreased peak intensity of MB dye confirms the degradation of MB dye by the Cu_2-X_Se catalyst. The degradation efficiency (%) of Cu_2-X_Se NPs against MB dye was calculated from Eqn. (10) as given below [47,51]:

Eqn. (10) $$Degradation\;\text{efficiency}\;\left(\%\right)=\frac{C_{0}-C_{t}}{C_{0}}*100$$

Where, C_0_ symbolizes initial absorbance and ‘C_t_’ shows the absorbance of dye after catalysis reaction in the presence of solar radiation. The percentages degradation of the MB dye solution at different catalyst concentrations and corresponding increase with increase in catalyst concentration are shown in Table 3. The degration with variation of catalyst concentration beyond 0.05 g is shown in Fig. 1 of supplementary data.

**Table 3:** Percentage degradation efficiency against MB dye solution with varying Cu_2-X_Se NPs concentrations and corresponding % increase in efficiency.

S. No.	Catalyst Concentration (g)	% degradation efficiency of MB dye in 120 min sun light exposure	% increase in degradation efficiency with catalyst concentration
**1**	MB without catalyst	0.00	0.00
**2**	0.01	24.47	24.47
**3**	0.02	45.25	20.78
**4**	0.03	60.95	15.70
**5**	0.04	81.27	20.32
**6**	0.05	90.30	9.03

### 3.7 Photo catalytic mechanism through Cu_2-X_Se NPs

The degradation capacity increases with increase in the Cu_2-X_Se NPs concentration from 0.01 g to 0.05 g of Cu_2-X_Se NPs as Beer-Lambert’s law said. The photo-degradation efficiency for 0.05g concentration shows optimum; 90.30 % against MB dye. The reason of degradation was that the catalyst Cu_2-X_Se NPs get adsorbed by the unsaturated bonds of dye molecules and converted them into saturated bonds; responsible for decolonization/degradation of MB dye. The schematic catalytic mechanism was shown in Fig. 4 (c) and possible chemical reactions of MB dye degradation (C_16_H_18_ClN_3_S) by Cu_2-X_Se NPs are shown below as:

Cu_2-X_Se + MB → (Cu_2-X_Se-MB) _ads_

(Cu_2-X_Se-MB) _ads_ + hϑ→ (Cu_2-X_Se-MB) ∗ _ads_

(Cu_2-X_Se-MB) ∗ _ads →_Cu_2-X_Se (e^−^_CB_ + h ^+^ _VB_)

Cu_2-X_Se (h^+^) + H_2_O → Cu_2-X_Se (OH•) + H^+^

Cu_2-X_Se (h^+^) + OH^−^_ads_ → Cu_2-X_Se (OH•)

O_2_ + e^−^→ O_2_^•^

O_2_^•^+ H^+^→ HO_2_

2HO_2 →_ H_2_O_2_ + O_2_^•^

H_2_O_2_ + O_2_^•^ → 2OH + O_2_

OH• + MB → Degraded Products

The MB dye with Cu_2-X_Se NPs solution exposed to the solar radiations, the electrons of Cu_2-X_Se NPs absorbed energy E ≥ 2.1eV produced an electron–hole pair (e^−^_CB_ + h ^+^ _VB_) and then leaving a hole in the valence band, electrons transferred to the surface of the catalyst. At the conduction band, electrons reduced the O_2_ of the solution to hydroxyl radicals, OH•. The holes of valance band react with H_2_O and form OH• radicals. The highly oxidizing OH• radicals were responsible for the degradation of MB dye. The Cu_2-X_Se NPs were responsible for the generation of a large number of free radicals, and then precipitated in the solution [[52], [53], [54], [55]]. one of the best thing about this work is that the photocatalytic dye degradation was studied via Cu_2-X_Se NPs without the use of catalytic reaction enhancers/reducing agents. The other researchers [21,[39], [40], [41], [42], [43]] used reducing agents such as NaOH and H_2_O_2_ during catalytic dye degradation as shown in Table 4.

**Table 4:** The Photo degradation efficiency and corresponding degradation time and used reaction enhancer agents during dye degradation.

S. No.	Composition	Degradation efficiency %	Degradation time (min)	Reaction enhancer agents during catalysis	Ref.
1	Cu_2-X_Se cubic	90.3	120 min	–	This work
2	CuSe Hexagonal	86.0	25 min	H_2_O_2_	[19]
3	Cu_3_Se_2_/Cu_2_Se/Cu_2_O hollow microsphere	90.0	100 min	H_2_O_2_	[21]
4	CuSe hexagonal	76.0, 87.0	90 min	H_2_O_2_	[38]
5	Cu_2_Se cubic	42.0	18 min	H_2_O_2_	[39]
6	CuSe hexagonal	74.0	360 min	–	[40]
7	Cu_2_Se cubic	98.2	60 min	NaOH, H_2_O_2_	[41]

### 3.8 Catalyst Re-usability (multi-cycle) studies

**Fig. 5:**
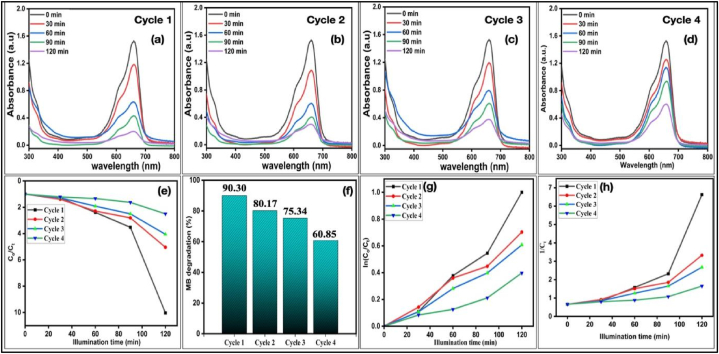
(a)–(d) absorption spectra of MB dye solution for re-usability test of sample for continuous four cycles, in one day spam, corresponding (e) degradation rate of MB dye in the presence of Cu_2-X_Se NPs (h) comparative degradation for four cycles of continuously re-used catalyst (f) pseudo first order photo-gradation kinetics rate (g) pseudo second order kinetics of Cu_2-X_Se NPs.

Since the optimum degradation efficiency (90.30 %) was obtained for 0.05g Cu_2-X_Se catalyst concentration in sunlight. Hence, 0.05g concentration was used for multi-cycle photocatalytic degradation analysis in continuation within one day spam to check the re-usability of synthesized Cu_2-X_Se NPs against MB dye. The continuous four cycles of photocatalytic degradation in one day spam were performed and obtained results were shown in Fig. 5(a–d). The results indicated the re-usability of Cu_2-X_Se NPs for photocatalytic degradation of MB dye with minor reduction in degradation capability. The degradation rates for four different cycles were shown in Fig. 5 (e) which predicted the catalysts Cu_2-X_Se NPs loses degradation capacity after each cycle. The degradation capacity of catalyst also influenced by the intensity of exposed sunlight because during the study in one day spam the sunlight exposure period having different sunlight intensities.The degradation efficiency decreases from 90.30 % to 80.17 % after 2nd cycle and then 75.34 %, & 60.85 % after 3rd and 4th cycle during of the continuous re-use of sample. The re-usability study was performed without reaction enhancers (reducing agents) during catalysis as shown in Table 5 and Fig. 5 (f). Table 5 shows that the random increase was observed in each 30 min duration change analysis. First three cycles showed the reduction in last 30 min duration in efficiency as 32.42 > 19.78> 15.32 but the fourth cycle show random increase in last 30 min of study. The photocatalytic analysis was performed in the exposure of sun light and the weather conditions can vary the intensity of sun light may be one of the reason of the variation in the degradation results during reusability analysis of catalyst.

**Table 5:** Degradation efficiency (%) for 0.05g concentration of Cu_2-X_Se NPs in 120 min’ exposure of solar radiations in different time hours of one day spam for re-usability analysis.

Time (min.)	Cycle 1		Cycle 2		Cycle 3		Cycle 4	
	% efficiency	% change	% efficiency	% change	% efficiency	% change	% efficiency	% change
0	0.0	0.0	0.0	0.0	0.0	0.0	0.0	0.0
30	22.25	22.25	28.15	28.15	21.99	21.99	17.69	17.69
60	58.18	35.18	56.30	28.15	47.72	25.73	25.20	7.51
90	71.59	13.41	64.39	8.09	60.02	12.03	38.61	13.41
120	90.30	32.42	80.17	19.78	75.34	15.32	60.85	22.24

### 3.9 Chemical kinetics

The chemical kinetics of photo degradation of MB dye with Cu_2-X_Se NPs were analysed using pseudo first order and second order models with time and shown in Fig. 5 (f & g). The first and second order reaction rate constants were calculated using Eqn. (11) for pseudo first order model of kinetics [56,57]; and Eqn. (12) for pseudo-second order reaction kinetics [58] given below as;

Eqn. (11) $$\log\frac{C_{0}}{C_{t}}=K_{1}t$$

Eqn. (12) $$\frac{1}{C_{t}}=k_{2}t+\frac{1}{C_{0}}$$

[C_0_]: Initial concentration of MB.

[C_t_]: Residuals concentration of MB

t: Time (min)

k_1_, k_2_: Rate constants for pseudo-first order (min^−1^) and pseudo-second order (M^−1^ min^−1^) respectively.

The calculated rate constants k_1_, k_2,_ and correlation coefficients R^2^ for multi-cycle degradation analysis were summarized in Table 6. Table 6 shows that correlation coefficients, R^2^ for pseudo-first-order was higher than pseudo-second-order and close to the unity. The multi-cycle degradation kinetics in the presence of sunlight for Cu_2-X_Se NPs were better described by pseudo-first-order model of degradation. Hence the multi-cycle catalysis results show that Cu_2-X_Se NPs are reusable for degrading organic pollutants from waste water.

**Table 6:** Experimental results of chemical kinetics rates for photo degradation of MB dye solution using 0.05g Cu_2-X_Se NPs catalyst for different models.

Successive Cycles	Pseudo first order model		Pseudo second order model	
	k_1_, min^−1^	R^2^	k_2_, min^−1^	R^2^
1	0.0187	0.9480	0.0447	0.7497
2	0.0131	0.9830	0.0209	0.8954
3	0.0116	0.9882	0.0161	0.9128
4	0.0071	0.9300	0.0075	0.8512

## 4 High resolution transmission electron microscope images

**Fig. 6:**
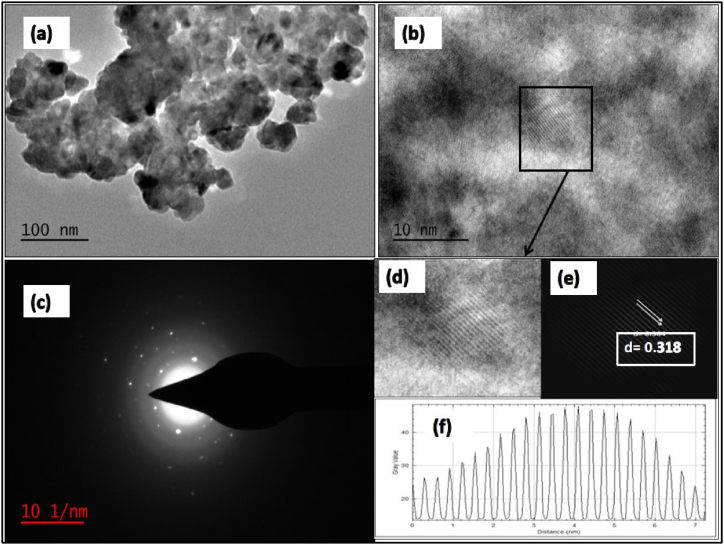
(a) TEM image (b) HRTEM image, (c) SAED pattern, (d) enlarged view of image (b), (e) Inverse FFT image of image (d), (f) profile plot of image (e) of Cu_2-X_Se NPs.

The TEM image, HRTEM image, SAED pattern and the interplanner spacing calculated by Image J software were shown in Fig. 6. The TEM image (a) clearly shows the formation of agglomerated NPs which were bunched together and of size around 20–25 nm and HRTEM image (b) shows the corresponding fine fringes. The SAED pattern of (c) exhibits the fused diffraction rings with bright spots confirmed that the sample is of good quality. The HRTEM image (b) was analysed with Image J software and image (d) is represented the enlarged view of image (b). The image (e) represented the inverse FFT image of (b) which is clearly shows the fine fringes and used to calculate fringe width ploting inverse FFT profile shown in image (e). The calculated fringe width or interplaner spacing is 0.318 which is in accordance to calculated interplanner spacing from XRD data of (111) planes (Table 1) of Cu_2-x_Se NPs.

## 5 Conclusion

In summary, highly crystalline Cu_2-X_Se NPs have been produced using an economic co-precipitation method. The XRD analysis confirmed the formation of cubic phase with crystallites size around 19 nm. The bending and stretching vibrations of Cu-Se bonds were confirmed by peaks at 716 cm^−1^ and 668 cm^−1^ of FTIR spectrum. The strong emission peak at 572 nm of the PL spectral region and corresponding CIE diagram showed that Cu_2-X_Se NPs were yellow light emitters. The SEM micrographs show that random shaped and sized grains are distributed throughout the sample surface and small grains staked over others to form large grains or patches. The EDX spectrum confirmed the presence of copper and selenium elements. The atomic ratios of copper and selenium (Cu: Se) are 1.69:1 confirms the formation of Cu_2-x_Se NPs where x = 0.31. The TEM images confirmed the formation of agglomerated NPs of size around 20 nm and the inter planar separation is 0.318 in accordance to the (111) plane of XRD. The optical band gap is 2.1eV; absorbs corresponding light from visible region of solar specrum for demonstrating excellent photo catalytic behavior against MB dye in aqueous solution. It is found that the degradation efficiency increases with increase in the catalyst concentration in the dye solution. The obtained optimum efficiency is 90.3 % for a 0.05 g catalyst concentration andthe continuous four-cycle sunlight driven re-usability analysis in a day spam is also shown with minor change in degradation efficiency. The corresponding degradation kinetics for 0.05g Cu_2-x_Se NPs were better described by pseudo-first-order model and the degradation occur by the interaction particle diffusion mechanism in the presence of sun light. Hence, Cu_2-X_Se NPs can be found appropriate for degrading organic pollutants from industrial waste water and other contaminated water bodies for the survival of aquatic life and, hence, for saving the environment.

## CRediT authorship contribution statement

**Pushpanjali Patel:** Writing – original draft, Software, Resources, Methodology, Investigation, Formal analysis, Conceptualization. **Rekha Garg Solanki:** Writing – review & editing, Supervision. **Prerna Gupta:** Formal analysis. **KM Sujata:** Formal analysis.

## Ethics approval

The work does not required ethical permission.

## Consent to participate

All authors have informed and given consent of participation.

## Consent for publication

All authors have informed and given consent of publication.

## Data availability

All data related to this article are included in the article and in supplymentry file.

## Funding

There is no funding source related to this work.

## Declaration of competing interest

The authors declare that they have no known competing financial interests or personal relationships that could have appeared to influence the work reported in this paper.
